# Safety and costs analysis of early hospital discharge after brain tumour surgery: a pilot study

**DOI:** 10.1186/s12893-020-00767-y

**Published:** 2020-05-14

**Authors:** Iuri Santana Neville, Francisco Matos Ureña, Danilo Gomes Quadros, Davi J. F. Solla, Mariana Fontes Lima, Claudia Marquez Simões, Eduardo Vicentin, Ulysses Ribeiro, Robson Luis Oliveira Amorim, Wellingson Silva Paiva, Manoel Jacobsen Teixeira

**Affiliations:** 1 Instituto do Cancer do Estado de São Paulo do Hospital das Clinicas da Faculdade de Medicina da Universidade de São Paulo, Av Dr Arnaldo 251 Cerqueira Cesar, CEP, São Paulo, 01246-000 Brazil; 2 Division of Neurosurgery, Hospital das Clinicas da Faculdade de Medicina da Universidade de São Paulo, Av Dr Arnaldo 251 Cerqueira Cesar, CEP, São Paulo, 01246-000 Brazil; 3 Division of Anaesthesiology, Hospital São Paulo, Universidade Federal de São Paulo, São Paulo, Brazil; 4 Financial, Planning, and Control Board, Instituto do Cancer do Estado de São Paulo do Hospital das Clinicas da Faculdade de Medicina da Universidade de São Paulo, São Paulo, Brazil; 5 Department of Gastroenterology, Faculdade de Medicina da Universidade de São Paulo, São Paulo, Brazil; 6 Universidade Federal do Amazonas, Manaus, Brazil

**Keywords:** Brain tumours, Discharge, ERAS, Enhanced recovery after surgery, Postoperative length of stay

## Abstract

**Background:**

A daily algorithm for hospital discharge (DAHD) is a key point in the concept of Enhanced Recovery After Surgery (ERAS) protocol. We aimed to evaluate the length of stay (LOS), rate of complications, and hospital costs variances after the introduction of the DAHD compared to the traditional postoperative management of brain tumour patients.

**Methods:**

This is a cohort study with partial retrospective data collection. All consecutive patients who underwent brain tumour resection in 2017 were analysed. Demographics and procedure-related variables, as well as clinical outcomes, LOS and healthcare costs within 30 days after surgery were compared in patients before/pre-implementation and after/post-implementation the DAHD, which included: stable neurological examination; oral feeding without aspiration risk; pain control with oral medications; no intravenous medications. The algorithm was applied every morning and discharge was considered from day 1 after surgery if criteria was fulfilled. The primary outcome (LOS after surgery) analysis was adjusted for the preoperative performance status on a multivariable logistic regression model.

**Results:**

A total of 61 patients were studied (pre-implementation 32, post-implementation 29). The baseline demographic characteristics were similar between the groups. After the DAHD implementation, LOS decreased significantly (median 5 versus 3 days; *p* = 0.001) and the proportion of patients who were discharged on day 1 or 2 after surgery increased (44.8% vs 3.1%; *p* < 0.001). Major and minor complications rates, readmission rate, and unplanned return to hospital in 30-day follow-up were comparable between the groups. There was a significant reduction in the median costs of hospitalization in DAHD group (US$2135 vs US$2765, *p* = 0.043), mainly due to a reduction in median ward costs (US$922 vs US$1623, *p* = 0.009).

**Conclusions:**

Early discharge after brain tumour surgery appears to be safe and inexpensive. The LOS and hospitalization costs were reduced without increasing readmission rate or postoperative complications.

## Background

Until early 1990s, perioperative care was based on empirical concepts and common practice, in part due to the paucity of scientific evidence. With the need of improving patient outcomes and reducing costs, the concern of developing safe and effective standards in postoperative care emerged, and advances have been achieved. In 1994, Engelmann and colleagues introduced the concept of “Fast-Track Surgery” to optimize postoperative recovery, reduce inpatient stay and related complications [1, 2]. In 2001, the creation of ERAS (Enhanced Recovery After Surgery) group represented a paradigm shift in protocols of care to patients undergoing gastrointestinal surgery [3]. In the following years, numerous guidelines were developed by other surgical fields (bariatric surgery, hepatectomy, head and neck cancer surgery, breast reconstruction surgery, among others), which are gradually transitioning to an outpatient-based paradigm [2, 4].

Even though many of these concepts are well stablished in literature, they are not widespread among neurosurgeons in their clinical practice. Traditionally, many neurosurgical centres still adopt an in-patient postoperative care with a median of 4 days after craniotomy (such as brain tumour resection and aneurysm clipping) for safety reasons, even in cases with no perioperative complications [5].

From 2008 to 2016, outpatient brain and spinal surgery experiences have been reported [6–8]. Despite the encouraging results, only after 2015 a significant number of publications on the subject containing neurosurgical patients have arisen, including one randomized clinical trial evaluating the implementation of the ERAS protocol [4, 9–11].

Recently, our institution, aiming to standardize hospital discharge, has adopted a daily algorithm for hospital discharge (DAHD), which is a key point in the concept of ERAS protocol. Thus, we designed a retrospective cohort study to evaluate whether there was a difference in length of stay (LOS), rate of complications, and hospital costs after the introduction of the DAHD compared to the traditional postoperative management of patients who underwent brain tumour resection.

## Methods

### Study design and patient population

This is a cohort study with partly retrospective data collection, conducted on a sequential period at the Instituto do Cancer do Estado de São Paulo (ICESP), University of Sao Paulo, Sao Paulo – Brazil. We have included all consecutive patients who underwent brain tumour resection during 2017 by a single neurosurgeon. Patients undergoing surgery for hydrocephalus, hematoma, cerebrospinal fluid leakage, surgical site infection, cranial reconstruction, endoscopic approach, and stereotactic biopsy were excluded so that we were focusing mainly on the subgroup of patients who underwent brain tumour resection by craniotomy. This retrospective patient medical record data review was approved by the local Ethics and Research Committee (CAAE 87064218.2.0000.0065).

### Daily algorithm for hospital discharge (DAHD)

**Fig. 1:**
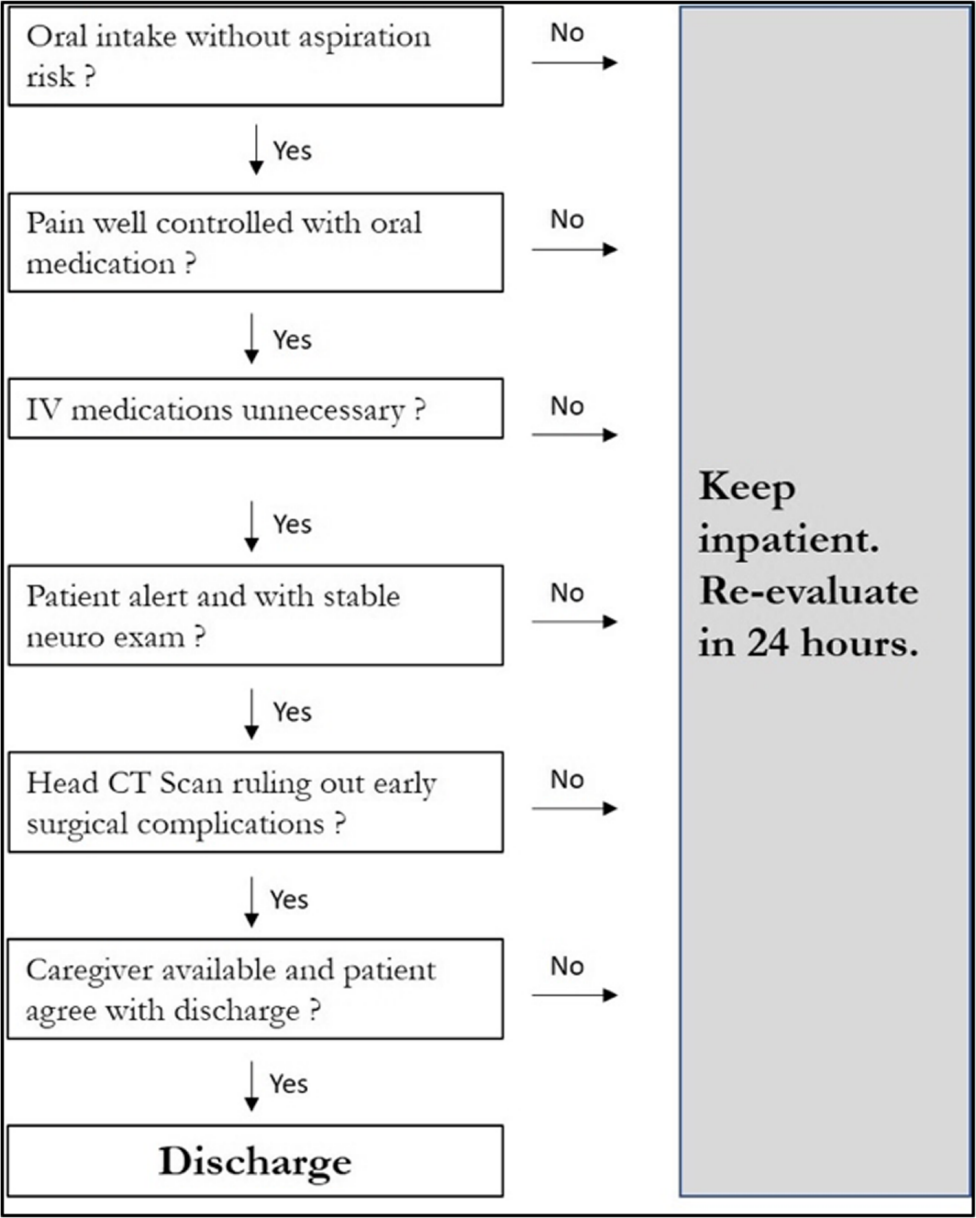
Daily Algorithm for Hospital Discharge (DAHD) - This algorithm includes all the criteria that each patient had to fulfill to be discharged. If any of these were not met, the participant was kept inpatient and re-evaluated the next day

The DAHD was recently introduced as an alternative to traditional management and classical discharge criteria of our institution. The DAHD (Fig. 1) is similar to the one proposed by Sughrue et al., 2015, [4] that includes: 1) neurological status before and after surgery; 2) Oral feeding without aspiration risk; 3) Pain control with oral medications; 4) Intravenous medications not needed; and 5) Stable neurological examination. The rationale of this algorithm was to consider hospital discharge from the day 1 after surgery if patient fulfilled the criteria. The algorithm was applied every morning during our routine round. If the patient did not meet a single criterion, he was kept inpatient and re-evaluated on the next day. Therefore, the patient was only kept inpatient if he objectively presented a real need for hospital care. Postoperative blood tests and head computed tomography were performed within 24 h and repeated as needed.

Two study groups were selected: 1) Pre-DAHD, retrospective, comprising patients who underwent brain tumour resection between January and August 2017, and 2) DAHD, which prospectively included patients operated between September and December 2017, after the implementation of the protocol.

### Anaesthetic and perioperative management

The perioperative management has not changed during the entire study period. All cases were discussed at the multidisciplinary tumour board meeting of our institution. Patients perform preoperative workup that includes basic laboratory tests, chest X-Rays, EKG, and preoperative evaluation by a general practitioner and an anaesthesiologist.

Preoperative fasting time of 8 h, and anti-thrombotic prophylaxis with graduated compression stockings and intermittent pneumatic compression, were always applied.

The type of anaesthesia, amount of fluid, and intraoperative management of neuromuscular blockade were left to the discretion of the attending anaesthesiologist and conventional total intravenous anaesthesia was used for general anaesthesia in patients who did not underwent awake craniotomy. Patients were administered a single dose of prophylactic antibiotics (second generation cephalosporin) 30 min before skin incision and scalp infiltration with 10 ml of 2% lidocaine was commonly used.

All patients underwent surgery by the smallest possible craniotomy that allowed a safe brain tumour removal. Perioperative brain mapping with navigated transcranial magnetic stimulation, neuronavigation, and intraoperative ultrasound were used when necessary.

The patients were preferably extubated in the operating room whenever possible and directly transferred to the postoperative intensive care unit (ICU). Patients were not extubated in the OR only if there were brain oedema leading to transcalvarian herniation after tumour removal, unexpected need to expand the craniotomy, or massive blood transfusion. Duration of surgery was not criteria to not extubate the patient. Postoperative dexamethasone and phenytoin doses were maintained as the preoperative schedule and weaning was managed case by case.

Oral feeding was reintroduced 6 hours after surgery for patients without dysphagia and with stable neurologic exam. Arterial lines, central venous catheters, and Foley bladder catheter were usually removed early the following morning (first postoperative day) and discharge from ICU was left to the discretion of the critical care staff physicians.

### Treatment costs

From each patient, all the costs comprising the surgery until the 30th postoperative day were collected. For this costs assessment, the absorption costing method was applied and included the costs of infrastructure and human and material resources used. For estimation of costs, the micro-costing method, which involves the direct measurement of every input consumed in the treatment of a particular patient, was adopted. The costs are shown in US dollars, converted from Brazilian reals (1US$ = R$3.846; Source: Central Bank of Brazil, October 24th – 2018)

### Data collection and statistical analysis

Patient demographic data, tumour characterization, general performance and neurological status, histologic diagnosis, complications, and clinical outcomes until 30 days after surgery were retrieved from patients’ medical records.

Categorical variables are presented as absolute frequencies and proportions and compared by means of the Pearson chi-squared or Fisher’s tests. The distribution of the continuous variables was verified by graphical and statistical (skewness and kurtosis) methods. These are presented as means and standard deviations or medians and quartiles and compared through the Student T test or Mann-Whitney test, as appropriate.

The primary outcome (length of stay) analysis was adjusted for the preoperative Karnofsky performance status (KPS) on a multivariable logistic regression model. We did not include post-operative complications on the multivariable adjustment.

All tests were two-tailed and final *p*-values < 0.05 were considered statistically significant. All analyses were performed on the software Statistical Package for Social Sciences, version 24.0 (SPSS, IBM Statistics, Armonk, NY, USA).

## Results

A total 66 patients were included, 32 on the Pre-DAHD and 34 on the DAHD group as depicted in Table 1. There were no differences between the two groups with respect to age, gender, BMI, ASA physical status, Karnofsky Performance Scale (KPS), and Eastern Cooperative Oncology group (ECOG) scores. Comorbidities such as hypertension, diabetes, and active smoking status were equally distributed between the two groups. In relation to preoperative clinical status, the majority of patients had no motor deficits, alteration of the level of consciousness, nor dysphagia.

**Table 1:** Characterization of the patient and preoperative clinical status

Variables	General (66)	Pre-DAHD (32)	DAHD (34)	p
**Age** (mean ± standard deviation)	51.5 ± 13,8	53.9 ± 12.5	49.2 ± 14.7	0.165 ^a^
**Female Gender**	39 (59.1)	14 (43.8)	13 (38.2)	0.649
**Ethnics**				0.854
Caucasian	32 (53.3)	15 (50.0)	17 (56.7)	
Brown (mulatto)	21 (35.0)	11 (36.7)	10 (33.3)	
African-American	7 (11.7)	4 (13.3)	3 (10.0)	
**Comorbidities**				
Body mass index (kg/m^2^)	25.1 (22.5–28.1)	25.1 (21.9–28.5)	25.0 (23.2–27.7)	0.495 ^b^
Obesity	11 (18.6)	5 (16.7)	6 (20.7)	0.692
Hypertension	23 (35.9)	12 (38.7)	11 (33.3)	0.654
Diabetes	7 (10.9)	3 (9.7)	4 (12.1)	1.000
Smoker	5 (7.8)	3 (9.7)	2 (6.1)	0.667
ASA	2 (2–3)	2 (2–3)	2 (2–3)	0.486
ASA ≥3	22 (33.8)	13 (40.6)	9 (27.3)	0.255
**Preoperative clinical status**				
Alteration of the LOC	10 (15.2)	7 (21.9)	3 (8.8)	0.180
Motor deficit				0.696
Not present	35 (53.0)	17 (53.1)	18 (52.9)	
Light (grade IV MRC)	16 (24.2)	9 (28.1)	7 (20.6)	
Severe (grade III MRC or less)	15 (22.7)	6 (18.8)	9 (26.5)	
Dysphagia	1 (1.5)	0 (0.0)	1 (3.0)	1.000
KPS < 70%	20 (30.3)	11 (34.4)	9 (26.5)	0.485
ECOG ≥3	14 (21.2)	9 (28.1)	5 (14.7)	0.183

Data presented as n (%), except for age (mean ± standard deviation) and body mass index (median and quartiles)

^a^ Student T test; ^b^ Mann-Whitney test

*ECOG* Eastern Cooperative Oncology Group, *KPS* Karnofsky Performance Scale, *LOC* level of consciousness, *MRC* Medical Research Council Scale

In respect to tumour characteristics and surgery performed, no significant differences between Pre-DAHD and DAHD groups were found (Table 2). High-grade glioma was the most common histology in Pre-DAHD group (*n* = 14, 43.8%), while metastasis comprised the majority of the patients of the DAHD group (*n* = 16, 47.1%), although not statistically significant (*p* = 0.455). The two groups were similar with relation to previous adjuvant treatment, number of lesions resected, deep seated lesions, infratentorial location, laterality, primary motor cortex involvement, presence of oedema, and cerebral herniation. The median of the larger lesion diameter was 47.5 (quartiles 32.3–58.0) and 41.0 (quartiles 30.0–52.5) millimetres in Pre-DAHD and DAHD groups, respectively (*p* = 0.345).

**Table 2:** Characterization of tumor and surgery

Variables	General (66)	Pre-DAHD (32)	DAHD (34)	p
**Tumor characteristics**				
Histology				0.455
Low grade glioma	11 (16.9)	4 (12.5)	6 (17.6)	
High-grade glioma	25 (38.5)	14 (43.8)	11 (32.4)	
Metastasis	28 (43.1)	13 (40.6)	16 (47.1)	
Meningothelial	1 (1.5)	0 (0.0)	1 (2.9)	
Inflammatory process	1 (1.5)	1 (3.1)	0 (0.0)	
Previous chemotherapy	22 (33.8)	9 (29.0)	13 (38.2)	0.434
Previous radiotherapy	12 (18.2)	7 (21.9)	5 (14.7)	0.450
Number lesions resected				
1	61 (92.4)	31 (96.9)	30 (88.2)	0.387
2	3 (4.5)	0 (0.0)	3 (8.8)	
3 or more	2 (3.0)	1 (3.1)	1 (2.9)	
Larger lesion diameter (mm)	44.5 (30.8–54.3)	47.5 (32.3–58.0)	41.0 (30.0–52.5)	0.345 ^a^
Infratentorial location	5 (7.6)	2 (6.3)	3 (8.8)	1.000
Depth > 1 cm of the cortex	33 (50.8)	16 (51.6)	17 (50.0)	0.897
Primary motor area involvement	15 (22.7)	7 (21.9)	8 (23.5)	0.873
Laterality				0.707
Right	25 (37.9)	10 (31.3)	15 (44.1)	
Left	34 (51.5)	18 (56.3)	16 (47.1)	
Bilateral	3 (4.5)	2 (6.3)	1 (2.9)	
Posterior Fossa	4 (6.1)	2 (6.3)	2 (5.9)	
Edema				0.064
Absent	15 (22.7)	4 (12.5)	11 (32.4)	
Little edema	29 (43.9)	15 (46.9)	14 (41.2)	
Very edema	22 (33.3)	13 (40.6)	9 (26.5)	
Herniation	35 (53.0)	15 (46.9)	20 (58.8)	0.331
**Characterization of surgery**				
Emergency surgery	5 (7.6)	2 (6.3)	3 (8.8)	1.000
Awake Surgery	3 (4.6)	1 (3.1)	2 (6.1)	1.000
Duration of surgery (min)	213 (165–291)	213 (155–298)	218 (169–283)	0.773 ^a^
Duration of anesthesia (min)	325 (285–414)	315 (281–429)	335 (289–403)	0.667 ^a^

Data presented as n (%), except for lesion diameter (medians and quartiles)

^a^ Mann-Whitney test

There was no significant difference between the two groups in terms of surgery characteristics: the median duration of surgery was 213 (quartiles 155–298) vs 218 (quartiles 169–283) minutes in Pre-DAHD and DAHD groups, respectively (*p* = 0.773). Emergency surgeries and awake craniotomies comprised the minority of the cases and were equally distributed in both groups.

**Fig. 2:**
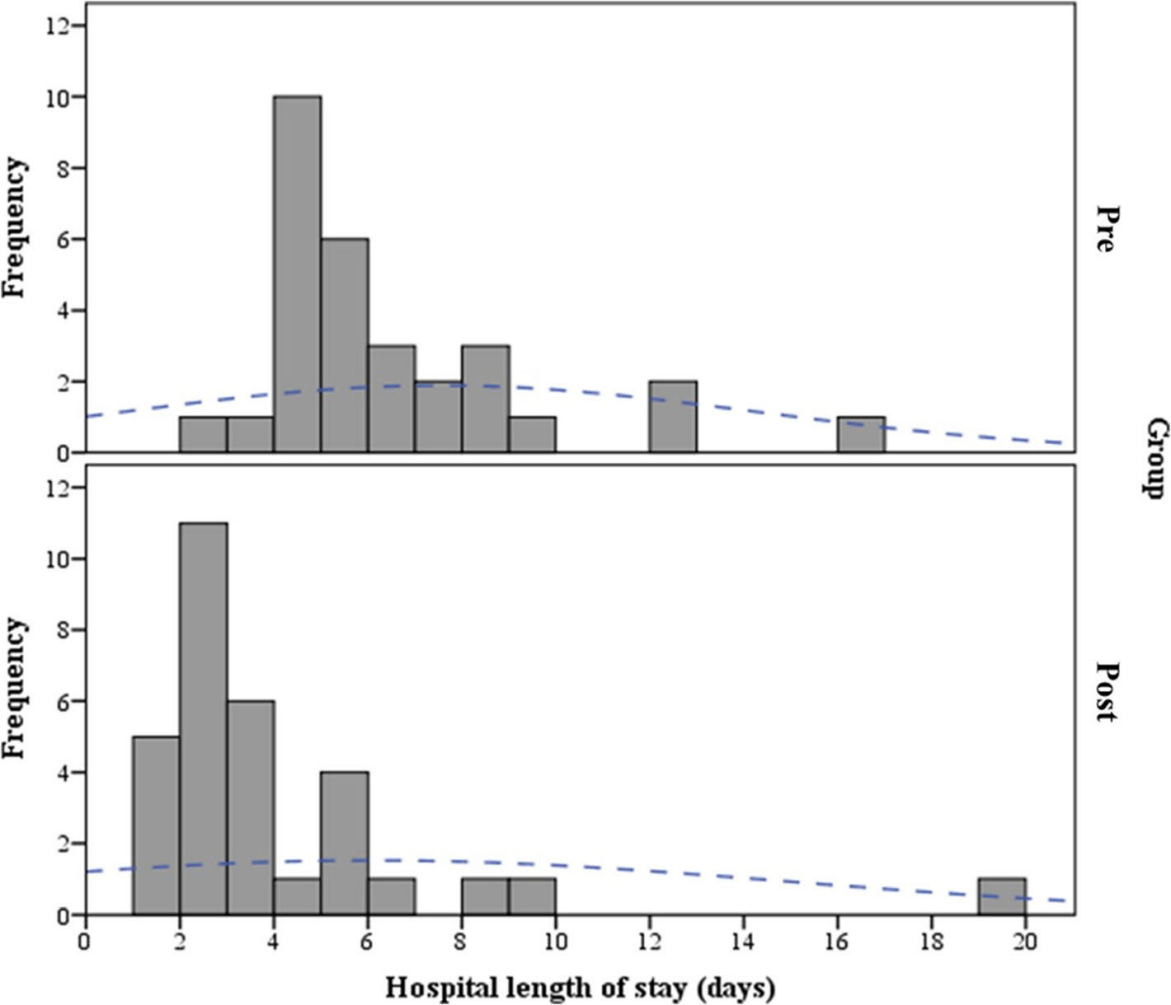
Histogram of length of hospitalization before (Pre-DAHD) and after DAHD implementation is shown above and below, respectively. There is an increase in the frequency of early (< 48 h) discharge after DAHD adoption

Relevant measured outcomes are shown in Table 3. In the majority of cases, gross total resection was achieved (78.8%) with no new motor deficits (67,7%) and these findings were similar between the two groups (*p* = 0.352 and *p* = 0.928, respectively). Following surgery, a higher percentage of patients in DAHD group were discharged within 48 h (47.1% vs 3.1%, *p* < 0.001). Conversely, the postoperative median length of stay (LOS) was lower in DAHD group at 3 (quartiles 2–5) days vs 5 (quartiles 4–8) days in Pre-DAHD group (*p* = 0.001) (Fig. 2). The median ICU stay was similar between the groups: 28.8 (quartiles 22.7–67.1) hours in Pre-DAHD and 26.5 (quartiles 23.5–50.9) hours in DAHD group (*p* = 0.663), although there was an increase in the proportion of LOS after surgery spent in ICU in DAHD group (0.44 [quartiles 0.33–0.65] vs 0.25 [quartiles 0.15–0.46], *p* = 0.030).

**Table 3:** Characterization of outcomes

Variables	General (66)	Pre (32)	Post (34)	p
Extent of resection				0.352
GTR	52 (78.8)	23 (71.9)	29 (85.3)	
STR	13 (19.7)	9 (28.1)	4 (11.8)	
Partial / Biopsy	1 (1.5)	0 (0.0)	1 (2.9)	
Postoperative motor status				0.928
Maintained	44 (67.7)	20 (64.5)	24 (70.6)	
Improvement	11 (16.9)	6 (19.4)	5 (14.7)	
New mild deficit	4 (6.2)	3 (9.7)	1 (2.9)	
New severe deficit	6 (9.2)	2 (6.5)	4 (11.8)	
Post-operative dysphagia	6 (9.2)	3 (9.7)	3 (8.8)	1.000
LOS				
Early discharge (%)	17 (25.8)	1 (3.1)	16 (47.1)	< 0.001
LOS (days)	4 (2–7)	5 (4–8)	3 (2–5)	0.001
ICU stay (hours)	26.6 (23.3–52.0)	28.8 (22.7–67.1)	26.5 (23.5–50.9)	0.663 ^a^
Proportion of ICU stay / LOS	0.38 (0.18–0.51)	0.25 (0.15–0.46)	0.44 (0.33–0.65)	0.030 ^a^

Categorical variables are presented as n (%) and continuous as medians and quartiles

^a^ Mann-Whitney test

*GTR* gross total resection, *ICU* intensive care unit, *LOS* Length of Stay, *STR* subtotal resection

The shorter LOS observed in DAHD group was maintained after multivariable adjustment for Pre-DAHD vs DAHD group, *p* = 0.002 (Table 4).

**Table 4:** Multivariate analysis by logistic regression to fit the patient’s performance

Variables	Coefficient ± SE	Wald	OR	CI 95%	***p*** value
DAHD (vs Pre-DAHD)	3.36 ± 1.09	9.61	28.92	3.45–242.78	0.002
KPS < 70 (vs ≥ 70)	−1.58 ± 0.88	3.22	0.21	0.04–1.16	0.073

*CI* Confidence interval, *DAHD* Daily algorithm for hospital discharge, *KPS* Karnofsky Performance Scale, *OR* Odds ratio, *SE* Standard error

Complications and safety outcomes are displayed in Table 5. Reoperations, major and minor complications, return do the emergency department, readmissions, and deaths until the 30th postoperative day were similar between the groups. All but two patients with minor or major complications were discharged beyond the 2nd POD, both cases with minor complications (one urinary tract infection and one had an isolated seizure on immediate post-operative period without any other neurological alterations). All the three cases of reoperations occurred in DAHD group, although this difference in incidence did not reach statistical significance (*p* = 0.239). None of the reoperations were due to late complications or beyond the 2nd POD. Severe sepsis was the most common cause of major complication in both groups, whereas seizures represented the main cause among the minor complication ones. Thirty-day return to the emergency department was very common in both groups: Pre-DAHD group – 7 (21.9%) patients and DAHD group – 10 (29.4%) patients, *p* = 0.484, which lead to six (18.8%) readmissions in Pre-DAHD and three (9.4%) in DAHD group, *p* = 0.474. Thirty-day mortality rate was 4.6% and did not differ between the groups: Pre-DAHD group – 1 (3.1%) patient vs DAHD group – 2 (6.1%) patients, *p* = 1.000.

**Table 5:** Complication and safety outcomes

Outcomes^**a**^	General (66)	Pre (32)	Post (34)	p
**Reoperations**	3^a^ (4.5)	0 (0.0)	3 (8.8)	0.239
Hematoma	2 (3.0)	0 (0.0)	2 (5.9)	0.493
Acute hydrocephalus	1 (1.5)	0 (0.0)	1 (2.9)	1.000
Brain edema	1 (1.5)	0 (0.0)	1 (2.9)	1.000
**Major complication**	10 (15.2)	6 (18.8)	4 (11.8)	0.505
Stroke	2 (3.0)	1 (3.1)	1 (2.9)	1.000
ACS	(0.0)	(0.0)	(0.0)	–
PT	1 (1.5)	1 (3.1)	0 (0.0)	0.485
Severe sepsis	8 (12.1)	5 (15.6)	3 (8.8)	0.469
Meningitis	2 (3.0)	2 (6.3)	0 (0.0)	0.231
State of epilepsy	2 (3.0)	2 (6.3)	0 (0.0)	0.231
**Minor complication**	11 (16.7)	6 (18.8)	5 (14.7)	0.660
Urinary tract infection	2 (3.0)	1 (3.1)	1 (2.9)	1.000
Pneumonia	(0.0)	(0.0)	(0.0)	–
Deep vein thrombosis	3 (4.5)	2 (6.3)	1 (2.9)	0.608
Surgical wound infection	1 (1.5)	1 (3.1)	0 (0.0)	0.485
Dehiscence	2 (3.0)	2 (6.3)	0 (0.0)	0.231
CSF Fistula	4 (6.1)	4 (12.5)	0 (0.0)	0.05
Seizures	7 (10.6)	4 (12.5)	3 (8.8)	0.705
**Return to the ED**	17 (25.8)	7 (21.9)	10 (29.4)	0.484
**Readmissions**	9 (14.1)	6 (18.8)	3 (9.4)	0.474
**Death**	3 (4.6)	1 (3.1)	2 (6.1)	1.000

The data is displayed as n (%)

^a^ One patient was reoperated because of hematoma and cerebral edema

**Legend:**^a^ Outcomes occurred until the 30th postoperative day. *ACS* acute coronary syndrome, *CSF* cerebrospinal fluid, *ED* emergency department, *PT* pulmonary thromboembolism

**Fig. 3:**
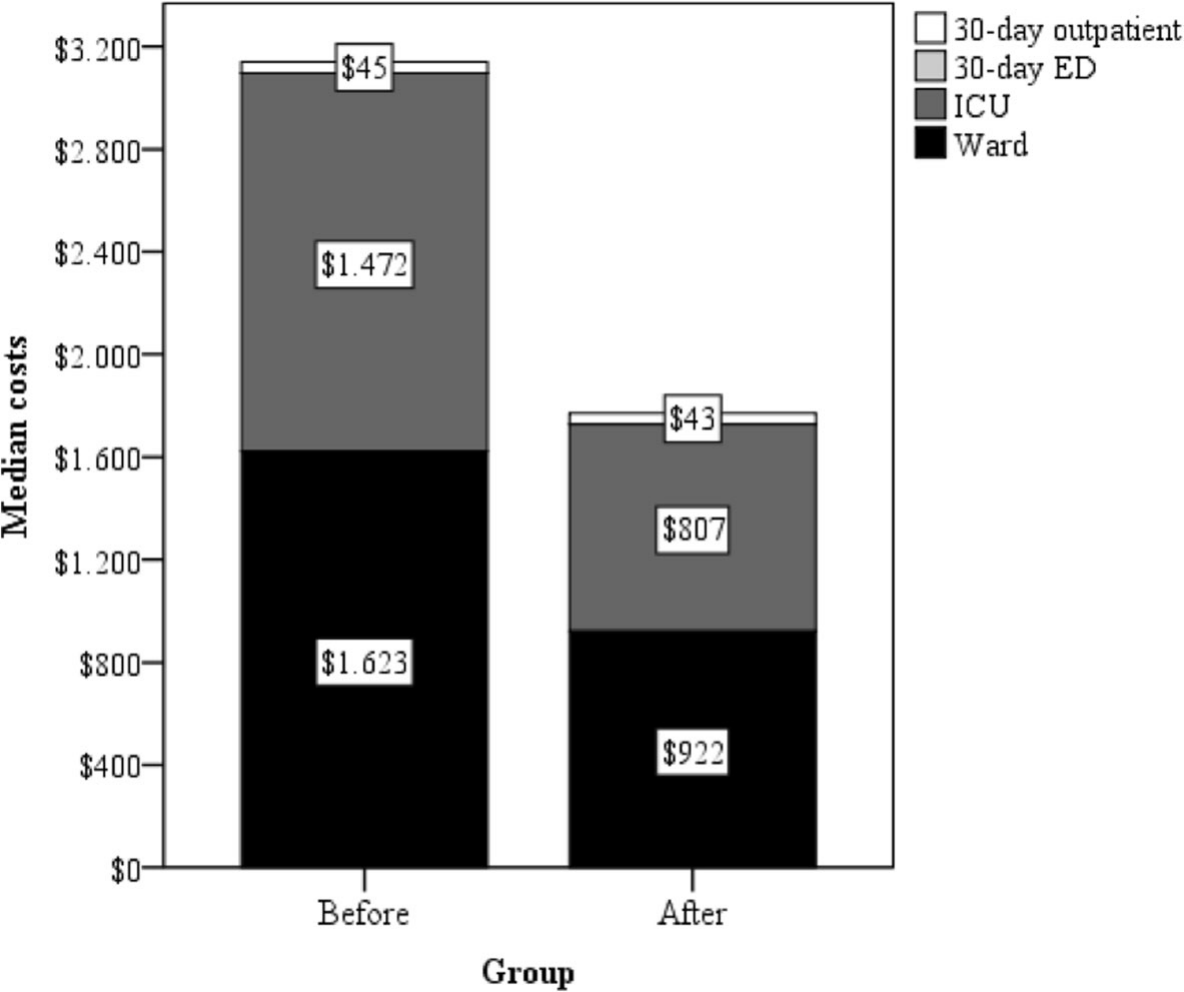
Distribution of 30-day inpatient and outpatient healthcare costs following brain tumor. This figure compares the distribution of 30-day healthcare costs between the two groups. Regarding ward costs, significant lower expenses were seen after DAH

Table 6 compares the costs of treatment between the two groups. There was a significant reduction in the costs of hospitalization in DAHD group (US$2135 [quartiles US$1472 – US$3800] vs US$2765 [quartiles US$2185 – US$4333], *p* = 0.043). Figure 3 depicts the distribution of costs in the two groups. While ICU costs were similar between the groups (*p* = 0.383), ward costs were significantly lower in DAHD group (US$922 [quartiles US$658 – US$1798] vs US$1623 [quartiles US$1155 – US$2507], *p* = 0.009). Thirty-day outpatient costs and 30-day overall costs were consonant between the two groups (*p* = 0.734 and *p* = 0.112, respectively).

**Table 6:** Costs of treatment between the two groups

Variables	General (66)	Pre (32)	Post (34)	p
**Costs of hospitalization**	2567 (1642–4137)	2765 (2185–4333)	2135 (1472–3800)	0.043
Ward	1436 (763–2311)	1623 (1155–2507)	922 (657–1798)	0.009
ICU	817 (741–1661)	1472 (747–2100)	807 (741–1614)	0.383
**30-day outpatient costs**				
Clinic	43 (35–87)	45 (0–85)	43 (40–87)	0.734
Emergency department	0 (0–537)	0 (0–0)	0 (0–613)	0.356
**Overall costs until 30th POD**	2963 (2041–4624)	3563 (2290–4915)	2511 (1796–4132)	0.112

**Legend**: Data are presented as median and interquartile interval in dollars (US$)

Intrahospitalar deaths were excluded from the analysis of outpatient costs

The Mann-Whitney test was used for all comparisons

*ICU* intensive care unit, *POD* postoperative day;

## Discussion

In this cohort of brain tumour patients, the adoption of the DAHD following surgery reduced postoperative LOS, with no increases in complication rates compared with traditional management after surgery.

Traditionally, all patients submitted to craniotomy for brain tumour resection in our institution were kept inpatient at least 4 days for neurological surveillance. This behaviour follows a worldwide trend in neurosurgical care, which has averages of intrahospital stays in the postoperative neurosurgical period ranging from 4 to 6 days [5, 12].

This study shed light on the shift in perioperative care with the adoption of quality improvement programs such as ERAS and Fast-track surgery and the need to evaluate the safety of these approaches in the surgical fields. We have adopted the DAHD in our Neuro-oncology department based on the experience of other brain tumour centre, [4] developed from the observation of an uncontrolled case series. Differently from the study that originated the DAHD, [4] we performed a historical cohort comparing two similar groups of patients, candidates for brain tumour resection before and after the implementation of DAHD. The high comparability between the groups gives greater reliability to our results and raises validity to the method.

Although there was a reduction of hospital LOS (median) from 5 days to 3 days after implantation of the protocol, the LOS in the ICU did not change. We believe that this is justified because all patients were assisted in the ICU bed after surgery and the assisting surgeons were not responsible for the clinical care adopted during this stay, so the algorithm could only be applied after the patient has been discharged from the ICU. Therefore, we believe that the hospital LOS could be even shorter if patients were assisted in a postoperative ward bed, which has been shown to be safe in other studies [6, 9]. In fact, routine outpatient care following brain tumour resection has been proved to be safe in selected patients [6, 10, 13], although patients with greater risks (large tumours, posterior fossa, worse status performance) were excluded. Sughrue et al., who included all patients with brain tumours at their institution, assisted patients in an intermediate care unit [4]. The use of protocols to identify patients at greater risk for complications or need for prolonged ICU stay (e.g. advanced age, diabetes, high intraoperative blood losses, and longer surgical procedures) could reduce the number of unnecessarily assisted ICU patients and may shorten the LOS [14, 15].

Healthcare costs were lower in the DAHD group during hospitalization mainly due to the reduction of nursing costs. Thirty-day costs, despite a tendency towards lower costs in the DAHD group, were not significant, although the current study may be underpowered for this outcome. Other studies that evaluated Fast-track protocols, due to a lack of control group, did not perform such comparisons [4, 6–9, 13].

Neurosurgical complications are quite common after brain tumour surgeries and these are the main reason for longer periods of LOS. The most common complications reported in the literature are venous thromboembolism, new or worsened neurological deficit, dural closure-related complications, postoperative peritumoural brain oedema, early postoperative seizure, general medical complications, wound infection, and surgery-related hematoma [5, 16, 17]. In our study, we had a higher incidence of complications (15.2% of *major* complications and 16.7% of *minor* complications) when compared to other similar case series, [4, 6–9, 11, 13] which may be due to many different factors: 1 - most of the studies selected patients with safer profiles for outpatient or early discharge surgery, and excluded patients with worsen performance status, large tumours or posterior fossa lesions; 2- this study was carried out in a tertiary referral hospital of a developing country, where patients submitted to neurosurgical treatment may have already severe neurological deficits, poor performance status, and greater volume of systemic disease (in cases of brain metastasis); 3 - our sample is composed mostly of malignant tumours (metastasis and high grade glioma comprised 81.6% of the sample), theoretically more prone to postoperative complications [18].

Reduction of costs has been pursued for many healthcare systems worldwide. Within this framework, shorter LOS and reduction in readmissions have been relevant outcomes. Thirty-day readmission rates have been used as a quality of healthcare indicator, with readmissions being associated with increased hospital costs and mortality [18–21]. We reported a high hospital readmission rate (14.1%), which may be explained by the large number of patients with poor performance status before neurological surgery (30% of patients with KPS < 70). However, complications, readmissions, and mortality rate were similar between the two groups, which supports that early discharge was safe and feasible even in our population profile.

There are several limitations of this study. This was a single-centre study with a small sample size, although our population was homogeneous. The study design, with cases and controls patients selected in different time frames, may contribute to biases, such as subtle increases of surgeon’s and multidisciplinary team experiences or other structural changes in the service that may have occurred during the different periods. Yet, the time frame of our study was short, which may have minimized such limitation. Moreover, seasonality issues and wash-out period, commonly applied and evaluated in before-after studies, were not investigated. Nevertheless, DAHD feasibility and safety were the main goals of this study. We believe that most patients and their relatives were fulfilled with early discharge, although it was not possible to retrospectively measure the level of satisfaction, which may be objective of future studies.

In 2018, Wang et al published the first prospective clinical trial evaluating the application of an ERAS protocol in cranial surgeries, showing significant benefits over conventional perioperative care, including reduction in postoperative LOS and faster recovery [11]. However, the adoption of this protocol required a large number of multidisciplinary professionals, including the need of a paradigm shift in the whole team, which makes their prompt reproducibility difficult to achieve in other settings. Our work provides feasible, not expensive small changes that can be adopted in any neurosurgical service before a greater multidisciplinary ERAS protocol could be adopted.

## Conclusion

The implementation of a standardized protocol for hospital discharge of patients submitted to brain tumour surgery led to a significant reduction in hospital LOS, reducing hospitalization costs, not increasing readmission rate or postoperative complications.

## Data Availability

The datasets used and/or analysed during the current study are available from the corresponding author on reasonable request.

## Abbreviations

DAHD: Daily Algorithm For Hospital Discharge
ECOG: Eastern Cooperative Oncology Group
ERAS: Enhanced Recovery After Surgery
ICU: Intensive Care Unit
KPS: Karnofsky Performance Status
LOS: Lenght Of Stay
SPSS: Statistical Package For Social Sciences
